# Genetic diversity of carotenoid-rich bananas evaluated by Diversity Arrays Technology (DArT)

**DOI:** 10.1590/S1415-47572009005000024

**Published:** 2009-01-30

**Authors:** Edson P. Amorim, Alberto D. Vilarinhos, Kelly O. Cohen, Vanusia B. O. Amorim, Janay A. dos Santos-Serejo, Sebastião Oliveira e Silva, Kátia N. Pestana, Vânia J. dos Santos, Norma S. Paes, Damares C. Monte, Ronaldo V. dos Reis

**Affiliations:** 1 Embrapa Mandioca e Fruticultura Tropical, Cruz das Almas, BABrazil; 2 Embrapa Recursos Genéticos e Biotecnologia, Brasília, DFBrazil

**Keywords:** diploids, germplasm, molecular markers, *Musa sp.*, variability

## Abstract

The aim of this work was to evaluate the carotenoid content and genetic variability of banana accessions from the *Musa* germplasm collection held at Embrapa Cassava and Tropical Fruits, Brazil. Forty-two samples were analyzed, including 21 diploids, 19 triploids and two tetraploids. The carotenoid content was analyzed spectrophotometrically and genetic variability was estimated using 653 DArT markers. The average carotenoid content was 4.73 μg.g ^-1^ , and ranged from 1.06 μg.g ^-1^ for the triploid Nanica (Cavendish group) to 19.24 μg.g ^-1^ for the triploid Saney. The diploids Modok Gier and NBA-14 and the triploid Saney had a carotenoid content that was, respectively, 7-fold, 6-fold and 9-fold greater than that of cultivars from the Cavendish group (2.19 μg.g ^-1^). The mean similarity among the 42 accessions was 0.63 (range: 0.24 to 1.00). DArT analysis revealed extensive genetic variability in accessions from the Embrapa *Musa* germplasm bank.

## Introduction

Fruit consumption has increased markedly in recent years, mainly because of its nutritional value and therapeutic effects. Fruits contain distinct phytochemicals, many of which have antioxidant properties that delay aging and prevent diseases, including certain types of cancer. Compounds such as β-carotenes and vitamins C and E are important antioxidant components of fruits (Wang *et al.*, 1997).

Bananas are widely consumed, primarily because of their low cost and their potential as functional and nutraceutical food. Brazil is the second largest banana producer in the world, with a production of 7.1 million tons in 2006 from ~500,000 hectares of plantations (FAO, 2008). Currently, commercial cultivars, especially those of the Cavendish group, do not contain significant amounts of substances with nutritional or therapeutic potential, such as polyphenols, vitamin C and carotenoids. In contrast, some banana genotypes identified in germplasm banks have high contents of these substances (Setiawan *et al.*, 2001; Someya *et al.*, 2002; Englberger *et al.* 2003a,b,c, 2005; Melo *et al.*, 2006; Wall, 2006; Davey *et al.*, 2007). Embrapa Cassava and Tropical Fruits has an active banana germplasm bank created through the introduction of local germplasm and from international collections. This germplasm bank consists of more than 400 accessions maintained in the field, including wild diploids, triploids and tetraploids.

Determination of the carotenoid content and genetic variability of these genotypes using DNA molecular markers can provide information that is useful in parental selection for crosses between divergent genotypes and for developing novel cultivars with functional properties. Several molecular markers, especially those associated with polymerase chain reaction (PCR)-based methods, including restriction fragment length polymorphism (AFLP), random amplified polymorphic DNA (RAPD) and microsatellites or simple sequence repeats (SSR), have been widely used to estimate genetic variability, choose genitors and investigate phylogenetic relationships among bananas (Creste *et al.*, 2003, 2004; Wan *et al.*, 2005; Ning *et al.*, 2007; Wang *et al.*, 2007; Jain *et al.*, 2007; Ruangsuttapha *et al.*, 2007).

Diversity arrays technology (DArT) is a novel genotyping technique that was originally developed for rice (Jaccoud *et al.*, 2001) but has been applied to cassava, wheat and *Arabidopsis thaliana* (Wittenberg *et al.*, 2005; Xia *et al.*, 2005; Stodart *et al.*, 2007; White *et al.*, 2008). DArT is an inexpensive technique that is based on hybridization and amplification, requires only a small amount of DNA and provided ample genome coverage (Stodart *et al.*, 2007).

The aims of this work were to quantify the total carotenoid content of 42 accessions of the banana germplasm bank held at Embrapa Cassava and Tropical Fruits, including diploids (AA), triploids (AAA, AAB and ABB) and tetraploids (AAAB), and to estimate the genetic variability by using DArT molecular markers to aid the choice of genitors for crosses done in the banana breeding program at Embrapa. To our knowledge, this is the first report on the use of DArT to evaluate banana diversity.

**Figure 1:**
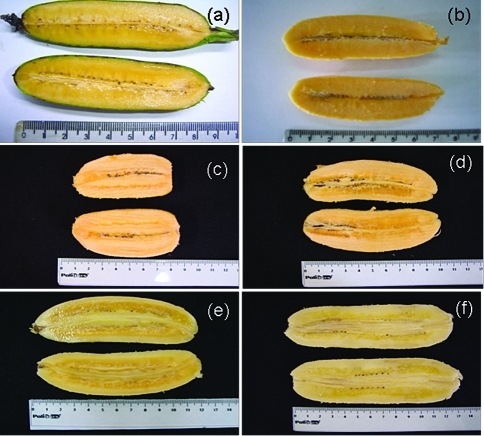
Banana accessions with a high carotenoid content and pulp color ranging from yellow to orange. Diploids: Modok Gier (a) and NBA-14 (b), triploids: Saney (c) and AAB no name (d), tetraploids: Ouro da Mata (e) and Porp (f).

**Figure 2:**
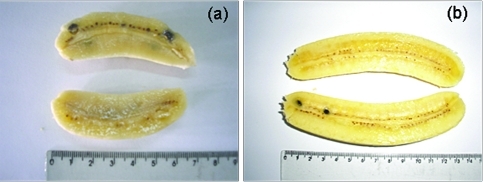
Banana accessions with a low carotenoid content and beige or white pulp color. (a) diploid Jambi and (b) triploid Markatooa. Detail: presence of seeds.

**Figure 3:**
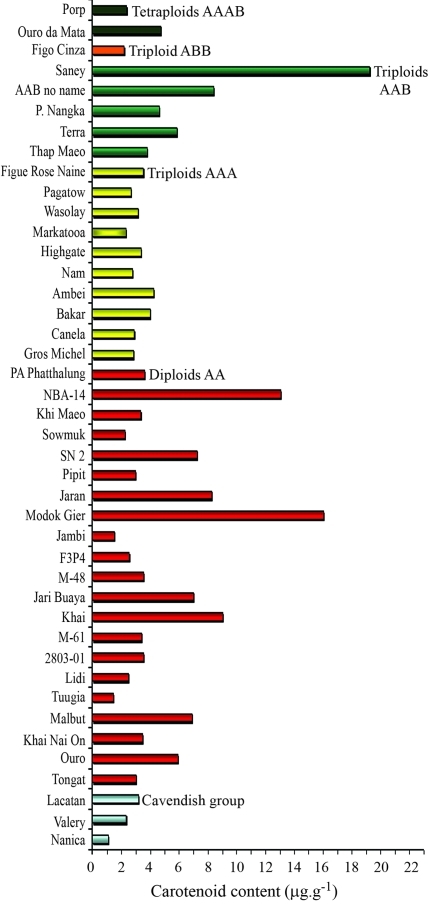
Carotenoid contents of 42 banana (*Musa*) accessions in the germplasm collection held at Embrapa Cassava and Tropical Fruits.

**Figure 4:**
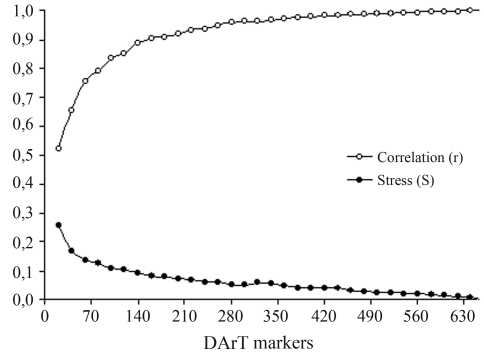
Bootstrap analysis used to obtain a precise estimate of the genetic similarity among 42 banana (*Musa*) accessions in the germplasm collection held at Embrapa Cassava and Tropical Fruits.

**Figure 5:**
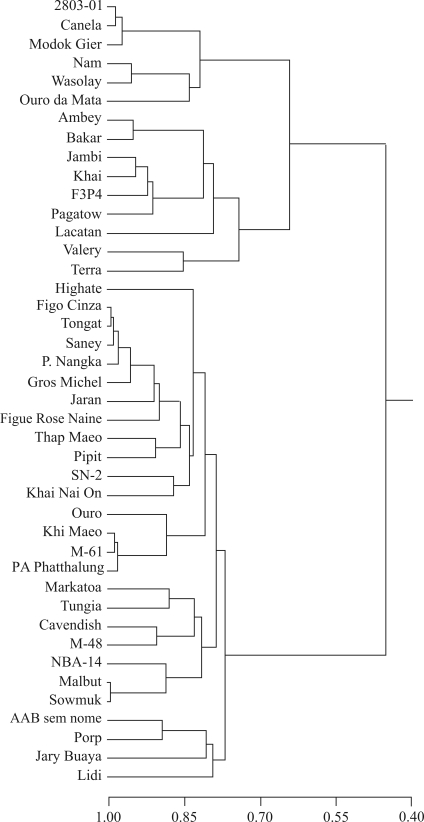
UPGMA dendrogram for 42 banana (*Musa*) accessions in the germplasm collection held at Embrapa Cassava and Tropical Fruits. The dendrogram was constructed with NTSYS version 2.0 software (Rohlf 2000) using the simple matching coefficient.

**Figure 6:**
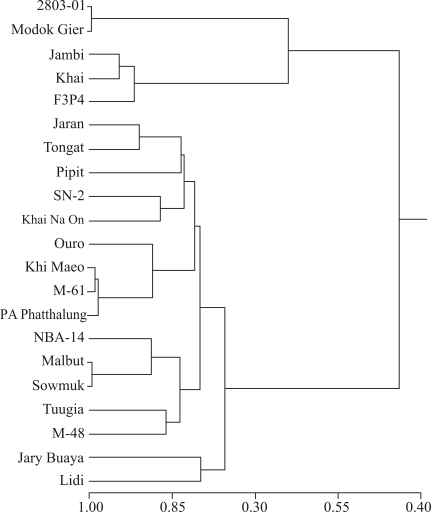
UPGMA dendrogram for the 21 diploid banana (*Musa*) accessions in the germplasm collection held at Embrapa Cassava and Tropical Fruits.  The dendrogram was constructed as described in Figure 5.

**Figure 7:**
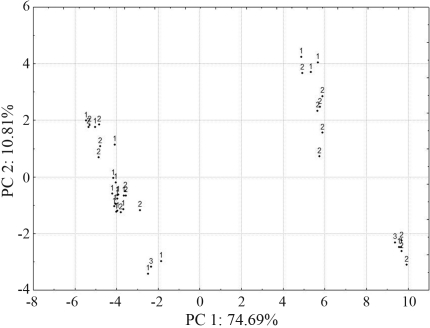
Clustering obtained by PCA based on pooled DArT data of 42 banana (*Musa*) accessions in the germplasm collection held at Embrapa Cassava and Tropical Fruits. 1 - diploids, 2 - triploids and 3 - tetraploids.

## Material and Methods

### Plant material

Forty-two banana accessions were used, including diploids (AA), triploids (AAA, AAB and ABB) and tetraploids (AAAB) maintained in the active banana germplasm bank at Embrapa Cassava and Tropical Fruits (Cruz das Almas, BA, Brazil). The accessions investigated were originally included in the germplasm bank based on their level of functional compounds such as total carotenoid content. The presence of nutraceutical compounds was recently introduced as an additional criterion for banana selection in breeding programs at Embrapa. Table 1 shows the level of ploidy, origin, color pulp, and carotenoid content of the accessions used in this work.

### Carotenoid analysis

The carotenoid content was determined spectrophotometrically, as described by Rodriguez-Amaya (1999). Banana samples (10 g) were macerated in a mortar containing celite and cold acetone and filtered through a sintered glass funnel. The extract was transferred to a separation funnel containing petroleum ether and ethylic ether (1:1, v/v) and the acetone:petroleum ether:ethylic ether mixture was washed repeatedly with distilled water the acetone was completely removed. The extract was dried with sodium sulfate, transferred to a suitable volumetric flask, and the volume was adjusted with petroleum ether. The carotenoid content was determined spectrophotometrically at 450 nm in a 1-cm cuvette. The samples were assayed in duplicate.

### Diversity analysis

#### DNA extraction 

Genomic DNA was extracted from leaves of the different genotypes by the cetyltrimethylammonium bromide (CTAB) method proposed by Doyle and Doyle (1990). After DNA extraction, the samples were stored in microtiter plates and shipped to Diversity Arrays Technology (DArT P/L, PO Box 7141, Yarralumba, ACT 2600, Australia), where the DArT analyses were done. The preparation of the *Pst*I*/Taq*I genomic representation DNA samples for DArT and the methodology for DArT, as provided by DArT P/L, are described below.

#### DArT procedure 

##### Restriction enzyme digestion and adapter ligation

A combined digestion/ligation reaction was prepared by adding 1 μL of DNA sample to 9 μL of digestion/ligation mix. The digestion/ligation reaction mixture contained 1 μL of 10X RE buffer (100 mM Tris-acetate (OAc), 500 mM KOAc, 100 mM Mg(OAc)_2_, 50 mM DTT, pH 7.8), 0.1 μL of BSA (New England Biolabs), 0.2 μL of 50 mM ATP, 0.1 μL of 5 μM *Pst*I adapter, 0.1 μL of *Pst*I (20 U/μL NEB), 0.1 μL *Taq*I (20 U/μL NEB), 0.2 μL of T4 DNA ligase (30 Weiss units/μL NEB) and 7.2 μL of ultrapure water. The samples were incubated at 37 °C for 90 min and at 60 °C for 90 min, heat inactivated at 80 °C for 20 min and stored at -20 °C until used.

#### PCR amplification of the genomic representation (target preparation)

PCR was used to create a genomic representation containing *Pst*I fragments that (i) showed no restriction sites for the frequent cutter used for redigestion and (ii) were short enough to be efficiently amplified. The number of PCR cycles was kept to a minimum to limit the bias towards fragments that were amplified more efficiently than others. The starting parameters for optimization are described below for the *Pst*I/*Taq*I method.

Forty-nine microliters of PCR mix were added to 1 μL of *Pst*I/*Taq*I digestion/ligation reaction used as the PCR template. The PCR mix consisted of (volumes per reaction): 5 μL of 10x PCR buffer (100 mM Tris-HCl pH 8.3, 500 mM KCl, 15 mM MgCl_2_, 0.1 % gelatin; Sigma), 1 μL of 10 mM dNTPs (Sigma), 2 μL of 10 μM *Pst*I+0, 2 μL of RED*Taq* (1 U/μL; Sigma) and 39 μL of ultrapure water. The PCR amplification program consisted of an initial step at 94 °C for 1 min, 30 cycles at 94 °C for 20 s, 58 °C for 40 s, and 72 °C for 1 min, and a final step at 72 °C for 7 min. Five microliters of PCR product were analyzed on 1.2% agarose gels to confirm that a homogeneous smear of fragments was obtained and to visualise the size distribution.

For hybridization and scanning, the targets were labelled with fluorescent dye (Cy3) and hybridized with the reference (Topo) labeled with FAM (Carboxyfluorescein) and hybridized to the array containing 6,000 DArT clones. Clone information cannot be made available because of data protection recommendations. After overnight hybridization at 62 °C, the arrays were washed and scanned at 20 μm resolution at 543 nm (Cy3) and 488 nm (FAM) on na LS300 confocal laser scanner (Tecan, Grödig, Austria).

### DArT data analysis

Array images were analyzed using DArTsoft 7.4 (Diversity Arrays Technology P/L, Canberra, Australia). The software automatically recognizes the array features using a seeded-region-growth algorithm and, for each fluorescent channel, reports the average and standard deviation (SD) of pixel intensities within and around each array feature, the fraction of saturated pixels, and the number of pixels within each feature, among other parameters. The logarithm of the ratio between the background-subtracted averages of feature pixels in the Cy3 and Cy5 channel (= log[Cy3target/Cy5reference]) was used as a measure of the difference in the abundance of the corresponding DNA fragment for the representations hybridized to an array. The values of log[Cy3/FAM] and log[Cy5/FAM], approximate measures of the amount of hybridization signal per amount of DNA spotted on the array, were used for quality control purposes.

Six hundred and fifty-three high-quality markers were scored. The results of the DArT analysis were expressed as a matrix (0-1). The mean genetic similarity among the genotypes was estimated by using a simple matching coefficient (Sokal and Michener, 1958). Cluster analysis was done by using the unweighted pair-group method with arithmetic average (UPGMA) as implemented in NTSYS-pc v. 2.1 (Rohlf, 2000). Principal component analysis (PCA)-based clustering with the sub-routine EIGEN was used to assess and visualize the level of ploidy (diploids, triploids and tetraploids) in diversity space (as opposed to visualizing only the genetic similarity among the accessions).

The cophenetic correlation coefficient was calculated and the Mantel test (Mantel, 1967) was used to check the fitness of a cluster analysis to the matrix on which it was based. Bootstrap analysis was used to verify the number of DArT markers needed to precisely determine the estimates of genetic similarity among the 42 banana accessions. This analysis was done with the statistical software GQMol (Cruz and Schuster, 2004) which estimates the correlation between values from the original distance matrix with those of other matrices obtained by re-sampling from different sample sizes (Amorim *et al.*, 2006). The software also calculates a value of stress (S) that indicates an adjustment between the original matrix and considers the 653 markers and re-sampling matrices (Kruskal, 1964).

## Results

Genotypes with a pulp color ranging from yellow to orange had a higher content of total carotenoids compared to those with white or beige pulp (Table 1 and Figures 1  and 2). Pulp color was found to be a suitable phenotypic criterion for making inferences about variability in the total carotenoid content of segregating populations. The mean content of total carotenoids among the 42 accessions was 4.73 μg.g^-1^, and ranged from 1.06 μg.g^-1^ for the triploid Nanica (Cavendish group) to 19.24 μg.g^-1^ for the triploid Saney (Table 1).

Within the diploid group, the mean carotenoid content was 5.24 μg.g^-1^, with high levels for the diploids Modok Gier (16.05 μg.g^-1^), NBA-14 (13.04 μg.g^-1^) and Khai (9.02 μg.g^-1^). The mean content among triploids from the genomic groups AAA, AAB and ABB was 2.93, 8.37 and 2.15 μg.g^-1^, respectively. The mean carotenoid content for tetraploids was 3.52 μg.g^-1^ (Table 1). Triploids with the genomic composition AAB had higher contents of total carotenoids compared to AAA triploids. In the Cavendish group of cultivars, the total carotenoid content was 1.06, 3.18 and 2.32 μg.g^-1^ for the Nanica, Lacatan and Valery genotypes, respectively, with a mean content of 2.19 μg.g^-1^. The carotenoid content of the diploids Modok Gier and NBA-14 and the triploid Saney was, respectively, 7-fold, 6-fold and 9-fold greater than the mean value for the Cavendish group (2.19 μg.g^-1^).

Figure 3 shows that there was extensive genetic variability in the carotenoid contents of the banana accessions, particularly among diploids. The three genotypes with the highest total carotenoid content (Modok Gier, NBA-14, and Saney) had the same geographic origin (New Guinea), indicating the possibility of gene flow among them. Our results indicate that it is possible to obtain segregating diploid populations for the trait ‘carotenoid content' that could be used to build genetic maps and identify quantitative trait loci (QTL) associated with the trait. In addition, by selecting suitable diploid and triploid genitors it is possible to develop tetraploid cultivars with high carotenoid contents.

Re-sampling analyses showed that 282 DArT markers (43% of the total) were sufficient to obtain a precise estimate of the genetic divergence among the 42 banana accessions. The correlation between the matrix considering all 653 markers and the matrix with 282 markers was 0.96, with a square sum of the deviations (SQ_d_) of 0.51 and a stress value (E) of 0.052 (Figure 4). These findings indicate that the number of DArT markers analyzed was sufficient to precisely estimate the genetic divergence among the 42 banana accessions. Moreover, ~60% of the DArT markers were inferred to have similar molecular information, probably as a result of genetic linkage among them in the banana genome. The mean genetic similarity among the 42 accessions was 0.63, and ranged from 0.24 between the diploid NBA-14 and the triploid Canela to 1.00 between the diploid 2803-01 and the accessions Canela (AAB) and Modok Gier (AA).

Figure 5 shows the dendrogram of genetic similarity obtained by UPGMA based on DArT among the 42 banana accessions investigated. The cophenetic value was high (r = 0.92, p < 0.0001, 10,000 permutations) and adequate since values of r ≥ 0.56 are considered ideal and reflect agreement with the values of genetic similarity (Vaz Patto *et al.*, 2004).

The cut-off value for the dendrogram was assumed to be the mean genetic similarity among all accessions genotyped by DArT (0.63). Based on this value, two major clusters were formed (Figure 5). The carotenoid contents showed no tendency to cluster since accessions with higher carotenoid levels occurred equally in both clusters and, in some cases, with low genetic similarity, *e.g.*, the diploids 2803-01 and NBA-14 (0.24). Similarly, DArT markers did not cluster the accessions based on their genomic group, *i.e.*, diploids (AA), triploids (AAA, AAB and ABB) and tetraploids (AAAB). A number of the accessions clustered based on geographic origin, whereas others showed no such correlation (Figure 5 and Table 1).

Figure 6 shows a dendrogram of the 21 diploid genotypes investigated by DArT markers. The average genetic similarity among the diploid accessions was 0.67, and ranged from 0.24 between NBA-14 and 2803-01 to 0.98 between Modok Gier and 2803-01. The accessions were clustered into three groups (Figure 5), although there was no clear separation between accessions with high and low carotenoid contents. The cophenetic value was high (r = 0.93, p < 0.0001, 10,000 permutations). As with the carotenoid content (Figure 3), DArT analysis also detected significant genetic variability among the 42 banana accessions maintained in the Embrapa germplasm bank (Figures 5  and 6).

Principal component analysis (PCA) reinforced the results obtained with clustering analysis by UPGMA (Figure 7). The first and the second components were responsible for 74.7% and 10.8% of the genetic variation, respectively. These two components were responsible for 85.5% of the variation observed among the levels of ploidy (diploids, triploids and tetraploids).

## Discussion

In this work, 42 banana accessions were initially screened based on pulp color, which ranged from yellow to orange. Some genotypes with white and beige pulp were also analyzed to confirm the association between pulp color and carotenoid content. Banana pulp color is directly related to carotenoid content and can be classified into five categories: white, beige, yellow, orangish-yellow and orange (Englberger *et al.*, 2003a,c). Hence, an orangish-yellow or orange pulp may be used as a criterion to select suitable genotypes to develop cultivars with a higher carotenoid content.

The mean carotenoid contents obtained here agreed with the results reported by others. Setiawan *et al.* (2001) reported that the mean β-carotene content of three samples of *Musa paradisiaca* was 1.00 μg.g^-1^ (range: 0.72 to 1.22 μg.g^-1^). Englberger *et al.* (2003b) quantified the total carotenoid content in 21 banana accessions, including genotypes from the sections Australimusa and Eumusa. The mean total carotenoid content was 11.13 μg.g^-1^, with values ranging from 0.60 to 53.7 μg.g^-1^. These authors reported a similar value for another 17 banana accessions (9.21 μg.g^-1^) (Englberger *et al.*, 2003c) and Melo *et al.* (2006) found a value of 10.62 μg.g^-1^ for the cultivar Comprida (Type Terra, AAB). Davey *et al.* (2007) reported that genotypes from the genomic group AAB had higher contents of carotenoids compared to those from the AAA group, possible because of the presence of the B genome. Similar results were reported by Englberger *et al.* (2003a) and Melo *et al.* (2006).

As shown here, the carotenoid content of some banana genotypes differed from that of Cavendish cultivars by up to 9-fold. In agreement with this, other studies of banana accessions in germplasm banks have reported carotenoid contents up to 25-fold higher than found in commercial cultivars from the Cavendish group (Englberger *et al.*, 2003a,b,c; Wall, 2006).

The carotenoid content may be influenced by several factors, including the degree of maturation, type of soil, climatic conditions, storage conditions, geographic location and, especially, genotype (Sharrock and Lutsy, 2000; Setiawan *et al.*, 2001; Souza *et al.*, 2004). In the present work, the most important factor was the genotype and the origin (geographic location) since all of the other factors were constant for the accessions in the Embrapa germplasm bank, *i.e.*, the accessions were analyzed at the same stage of maturation, stored under the same conditions and kept under the same environmental conditions.

Re-sampling analyses indicated that the number of DArT markers used was more than sufficient to precisely genotype the 42 banana accessions. The stress value (E) of 0.052 reinforced this conclusion, since E values ≤ 0.05 are considered ideal for precise estimation of the genetic variation in a given population (Kruskal, 1964).

Extensive genetic variability was observed among the 42 accessions genotyped using DArT. Similar results were obtained by Creste *et al.* (2003, 2004) who used microsatellite or SSR markers to evaluate diploid and triploid accessions from the Embrapa germplasm bank. The lack of genetic correlation between accessions of common genetic origin is probably related to recent anthropic action that has influenced genotype dispersion (Jarret *et al.*, 1993).

In the present study, several triploid cultivars shared high similarity values with diploid genotypes, *e.g.*, the triploid Nanica, which showed 91% similarity with the diploid M-48, 84% similarity with the diploids Khai Nai On and Tuugia and 80% similarity with the diploids Sowmuk, Jaran and NBA-14. Since Nanica is an export-type triploid cultivar (Cavendish) and exhibits complete female sterility, the high level of similarity with diploids provides novel breeding possibilities.

Molecular analyses have revealed extensive genetic diversity in *Musa acuminata* (Lannaud *et al.*, 1992; Carreel *et al.*, 1994, 2002; Ude *et al.*, 2002; Ruangsuttapha *et al.*, 2007). In contrast, analyses using RAPD, SSR and AFLP molecular markers have revealed lower levels of genetic variation among banana accessions (Nsabimana and Staden, 2007; Ning *et al.*, 2007; Wang *et al.*, 2007). In this context, the use of DArT markers to clearly define the relationships among and within wild material and to improve diploid and triploid cultivars in germplasm collections could facilitate the prediction of how hybrids will perform, thereby increasing genetic gains.

Our results provide additional information for banana genetic breeding programs by allowing the selection of carotenoid-rich diploids for crosses with triploids to provide novel tetraploid cultivars with improved functional properties. Nutritional (biofortification) and functional breeding, allied with improved productivity and disease resistance, should result in better nutritional value. Since bananas are the most consumed fruit in the world, the ingestion of nutritionally enhanced cultivars could significantly improve consumers health by reducing the incidence of diseases and indirectly decrease spending on public health, which is currently significantly elevated worldwide.

## Figures and Tables

**Table 1:** Carotenoid content of 42 accessions from the banana (*Musa*) germplasm collection at Embrapa Cassava and Tropical Fruits.

Accession / ITC	Ploidy	Origin	Pulp color	CC	Accession	Ploidy	Origin	Pulp color	CC
Modok Gier	AA	New Guinea	Orange	16.05 ± 0.41*	Ambei	AAA	New Guinea	Orangish-yellow	4.22 ± 0.29
NBA 14 / 0267	AA	New Guinea	Orange	13.04 ± 0.05	Bakar / 1064	AAA	Indonesia	Yellow	3.98 ± 0.62
Khai / 0532	AA	Thailand	Orangish-yellow	9.02 ± 0.22	Figue Rose Naine / 1159	AAA	France	Yellow	3.50 ± 0.32
Jaran / 0678	AA	Indonesia	Orangish-yellow	8.23 ± 0.79	Highgate / 0263	AAA	Honduras	Yellow	3.34 ± 0.51
SN2	AA	New Guinea	Yellow	7.23 ± 0.71	Lacatan / 0768	AAA	France	Yellow	3.18 ± 0.16
Jari Buaya / 0312	AA	Honduras	Yellow	7.01 ± 0.78	Wasolay	AAA	New Guinea	Yellow	3.15 ± 0.17
Malbut	AA	New Guinea	Yellow	6.87 ± 0.24	Canela	AAA	Brazil	Yellow	2.86 ± 0.44
Ouro	AA	Brazil	Yellow	5.91 ± 0.38	Gros Michel / 0484	AAA	Brazil	Cream	2.81 ± 0.67
PA Phatthalung	AA	Thailand	Yellow	3.59 ± 0.34	Nam / 1303	AAA	Thailand	Yellow	2.77 ± 0.14
2803-01	AA	Brazil	Yellow	3.53 ± 0.14	Pagatow	AAA	New Guinea	Yellow	2.64 ± 0.25
M-48	AA	Equador	Yellow	3.51 ± 0.19	Valery / 0048	AAA	Indonesia	Yellow	2.32 ± 0.24
Khai Nai On / 0532	AA	Thailand	Yellow	3.42 ± 0.23	Markatooa	AAA	New Guinea	White	2.29 ± 0.25
M-61	AA	Equador	Yellow	3.37 ± 0.52	Nanica	AAA	Brazil	White	1.06 ± 0.04
Khi Maeo	AA	Thailand	Yellow	3.32 ± 0.15	P. Nangka / 0004	AAB	Thailand	Yellow	4.59 ± 0.01
Tongat / 0063	AA	Honduras	Yellow	3.01 ± 0.31	Saney	AAB	New Guinea	Orange	19.24 ± 3.22
Pipit / 0685	AA	Indonesia	Beige	2.95 ± 0.14	AAB no name	AAB	Honduras	Orange	8.40 ± 0.34
F3P4	AA	Equador	Yellow	2.52 ± 0.03	Terra	AAB	Brazil	Orangish-yellow	5.83 ± 1.16
Lidi / 0395	AA	Honduras	Beige	2.45 ± 0.08	Thap Maeo / 1301	AAB	Honduras	Cream	3.77 ± 0.04
Sowmuk / 0266	AA	New Guinea	Yellow	2.23 ± 0.07	Figo Cinza	ABB	Brazil	Yellow	2.15 ± 0.01
Jambi	AA	Indonesia	Beige	1.47 ± 0.01	Ouro da Mata / 0261	AAAB	Brazil	Orangish-yellow	4.70 ± 0.02
Tuugia	AA	Hawaii	White	1.41 ± 0.04	Porp	AAAB	New Guinea	Beige	2.34 ± 0.06

ITC: International Transit Centre. CC: carotenoid content (μg.g^-1^). *: means (±standard error) of two replicates per accession.
